# Sensorless Self-Excited Vibrational Viscometer with Two Hopf Bifurcations Based on a Piezoelectric Device

**DOI:** 10.3390/s21041127

**Published:** 2021-02-05

**Authors:** Shinpachiro Urasaki, Hiroshi Yabuno, Yasuyuki Yamamoto, Sohei Matsumoto

**Affiliations:** 1 Graduate School of System and Information Engineering, University of Tsukuba, Tsukuba, Ibaraki 305-8573, Japan; urasaki.shimpachi.xu@alumni.tsukuba.ac.jp; 2 Liquid Flow Standards Group, Research Institute for Engineering Measurement, National Metrology Institute of Japan (NMIJ), National Institute of Advanced Industrial Science and Technology (AIST), Tsukuba, Ibaraki 305-8563, Japan; yamamoto-yasu@aist.go.jp; 3 Device Technology Research Institute, National Institute of Advanced Industrial Science and Technology (AIST), Tsukuba, Ibaraki 305-8564, Japan; sohei.matsumoto@aist.go.jp

**Keywords:** viscometer, sensorless self-excited oscillation, piezoelectric device, Hopf bifurcation, double Hopf bifurcation, jump phenomenon

## Abstract

In this study, we propose a high-sensitivity sensorless viscometer based on a piezoelectric device. Viscosity is an essential parameter frequently used in many fields. The vibration type viscometer based on self-excited oscillation generally requires displacement sensor although they can measure high viscosity without deterioration of sensitivity. The proposed viscometer utilizes the sensorless self-excited oscillation without any detection of the displacement of the cantilever, which uses the interaction between the mechanical dynamics of the cantilever and the electrical dynamics of the piezoelectric device attached to the cantilever. Since the proposed viscometer has fourth-order dynamics and two coupled oscillator systems, the systems can produce different self-excited oscillations through different Hopf bifurcations. We theoretically showed that the response frequency jumps at the two Hopf bifurcation points and this distance between them depends on the viscosity. Using this distance makes measurement highly sensitive and easier because the jump in the response frequency can be easily detected. We experimentally demonstrate the efficiency of the proposed sensorless viscometer by a macro-scale measurement system. The results show the sensitivity of the proposed method is higher than that of the previous method based on self-excited oscillation with a displacement sensor.

## 1. Introduction

Viscosity is an essential parameter frequently used in many fields such as food, petrochemistry, and biology. While a variety of viscosity sensors have been proposed [1,2], vibrational sensors are receiving special attention because they provide instantaneous and continuous readings of the target viscosity as it changes with time [3,4,5]. In recent years, there has been increasing need to determine the rheological properties of cells, blood, and other biological matter [6,7,8,9,10]. Hence, miniaturized sensing probes such as the micro-cantilever [11,12,13,14], fabricated with micro-electro-mechanical system (MEMS) technology, are used for those measurements. Many sensing principles have been proposed to meet these requirements.

The three existing excitation methods for a vibrational viscosity sensor are: frequency response under external forcing, feedback control with a sensor, and using the electric impedance of equivalent circuits. The most basic method is based on the frequency response under external forcing, which uses the fact that a higher viscous environment blunts the shape of the resonator’s frequency response curve. This method detects viscosities from the quantitative change in the peak frequency or the quality factor [15,16,17,18,19]. The quality factor, or Q-value, indicates the sharpness of the curve. The external excitation method is very simple, but its accuracy and sensitivity may deteriorate in high-viscosity sensing because the peak of the frequency response curve cannot be identified accurately. Moreover, the method is not applicable to much higher viscosities where the resonance peak does not appear in the frequency response curve. The second method, based on feedback control using oscillating velocity feedback [20,21,22], a phase-locked-loop (PLL) [23], and a phase shifter [24,25], can overcome this difficulty because it directly determines the viscosity without using the frequency response curve.

In the first and second methods, it is essential to use a sensor such as a laser displacement sensor or laser Doppler vibrometer (LDV). The third method, based on equivalent circuit models and impedance analysis, does not require a sensor. Equations of motion expressed with mechanical parameters such as the mass, stiffness, and viscous damping characteristic of the resonator can be equivalently converted to circuit equations expressed with electrical parameters such as the capacitance, inductance, and electric resistance. The Butterworth-Van Dyke (BVD) model is often used as an equivalent model for mass or viscosity sensors [26,27,28,29,30]. Because these methods determine the viscosity from the frequency characteristic of the impedance, they cannot overcome the difficulty in the first method. Furthermore, because impedance is influenced by not only mechanical properties but also circuit properties such as the resistance of the electro-probe itself, a change in viscosity may not provide enough change in impedance.

In this study, we propose a new viscosity measurement method with sensorless self-excited oscillation to enhance the sensitivity and usability of micro-fabrication systems. Our research group has developed sensorless self-excited oscillation for cantilevers, that do not require any detection of the displacement or velocity of the resonator [31]. This sensorless self-excited oscillation uses the interaction between the mechanical dynamics of the cantilever and the electrical dynamics of the piezoelectric device attached to the cantilever. The current supplied to the piezoelectric actuator consists of components proportional to the voltage of the piezoelectric device and to its differential. There are existing uses of sensorless self-excited oscillation, but it has not been applied to viscosity measurement.

For application to viscosity measurement, we focus on jumps in the response frequency. The proposed sensorless self-excited oscillation viscometer has fourth-order dynamics and two coupled oscillator systems for feedback control. The low-pass filter included in the feedback control was used not only for noise elimination in [31], but also as a control parameter to detect the viscosity. In cases when the cut-off frequency of the filter is swept forward or backward, the systems can produce different self-excited oscillations through different Hopf bifurcations. Then, we find the relationship between the width of the hysteresis and the viscosity. This enables highly sensitive measurements because the width of the hysteresis can be easily determined from jumps in the response frequency in the sweeps. We theoretically derive the condition for the existence of hysteresis and the relationship between the width of the hysteresis and the viscosity. Furthermore, we experimentally demonstrate the efficiency of the proposed sensorless viscosity measurement system using a macro-scale cantilever with a bimorphic piezoelectric device.

## 2. Principle of Viscosity Measurement Based on Sensorless Self-Excited Oscillation

### 2.1. Theoretical Modeling of the Sensorless Viscometer System

#### 2.1.1. Analytical Model and Governing Equations

**Figure 1:**
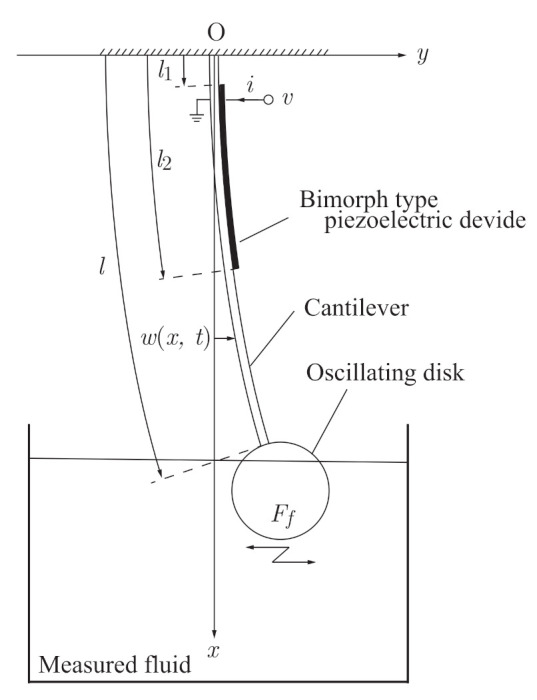
Analytical model of sensorless self-excited vibrational viscometer driven by piezoelectric device. The oscillating thin rigid disk is subjected to fluid force $F_{f}$ from the measured fluid.

We introduce the analytical model of the sensorless viscometer shown in Figure 1. The system consists of a cantilever with a bimorphic piezoelectric device, whose free end has a rigid thin disk. The disk is immersed in a Newtonian sample fluid and subject to fluid force $F_{f}$. The mass of the disk is small enough for us to neglect its inertial and gravitational forces, while the fluid force acting on the disk affects the dynamics of the cantilever.

The *x* and *y* axes denote the downward direction from the fixed end of the cantilever and the lateral direction, respectively. The ends of the bimorphic piezoelectric device are attached at distances $l_{1}$ and $l_{2}$ from the fixed end of the cantilever. The quantities w(x,t), v(t), and i(t) are the flexure of the cantilever, the voltage across the terminals of the piezoelectric device, and the current supplied to the piezoelectric device, respectively. Additionally, w(x,t) can be expressed as the product of a function depending on time *t* and one depending on vertical coordinate *x*: $w\left(x,t\right)=\sum_{i=1}^{\infty }a_{i}\left(t\right)\Phi_{i}\left(x\right)$, where $\Phi_{i}\left(x\right)$ and $a_{i}\left(t\right)$ are the *i*th modal function and the corresponding time-dependent displacement, respectively. By assuming that the cantilever oscillates with the first mode and projecting the flexure w(x,t) onto the first mode according to [21,31], we obtain the governing equations as

(1) $$\begin{array}{c} m\frac{d^{2}a_{1}}{dt^{2}}+\left(c_{0}+c_{f}\right)\frac{da_{1}}{dt}+\left(k_{0}+k_{f}\right)a_{1}=-\psi v, \end{array}$$

(2) $$\begin{array}{c} \frac{dv}{dt}=\frac{1}{C_{p}}i+\frac{\psi }{C_{p}}\frac{da_{1}}{dt}, \end{array}$$

where $a_{1}$ is the displacement of the cantilever in the first mode. Equations (1) and (2) describe the dynamics of the first-mode cantilever oscillation and the electric circuit of the piezoelectric device, respectively. The first, second, and third terms on the left side of Equation (1) express the inertial, damping, and restoring forces, respectively, and the term on the right side of Equation (1) is the control input of the piezoelectric actuator. The mass, damping, and bending stiffness of the cantilever itself are denoted by the terms that include *m*, $c_{0}$, and $k_{0}$, respectively, and the fluid force $F_{f}$ is represented by the terms with $c_{f}$ and $k_{f}$ [20,21]. The quantity ψ on the right side of Equations (1) and (2) is the electro-mechanical coupling coefficient between the cantilever and the piezoelectric device, and $C_{p}$ on the right side of Equation (2) is the capacitance of the piezoelectric device [31]. Because the mechanical dynamics of the cantilever and the electrical dynamics of the piezoelectric device influence each other via the coupling coefficient ψ in Equations (1) and (2), the entire system is regarded as a third-order coupling system.

#### 2.1.2. Proposed Feedback Control

**Figure 2:**
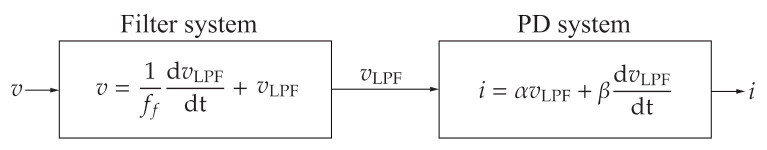
Illustration of the feedback controller. The feedback controller is constructed as the cascade connection of a first-order filter system and PD (proportional-derivative) feedback system. *v* and *i* denote the voltage across the terminals of the piezoelectric device and the current supplied to the piezoelectric device, respectively. The voltage *v* and current *i* are input and output signals, respectively, and $v_{\mathrm{LPF}}$ is the output signal of the filter system.

We propose the following feedback controller to produce the self-excited oscillation without any sensors:

(3) $$\begin{array}{cc} \; & \frac{1}{f_{f}}\frac{dv_{\mathrm{LPF}}}{dt}+v_{\mathrm{LPF}}=v, \end{array}$$

(4) $$\begin{array}{cc} \; & i=\alpha v_{\mathrm{LPF}}+\beta \frac{dv_{\mathrm{LPF}}}{dt}, \end{array}$$

which is schematically shown in Figure 2. The input and output signals of the controller are the voltage across the terminals of the piezoelectric device, *v*, and the current supplied to the piezoelectric device, *i*. Then, the original third-order coupling system is transformed into a fourth-order coupling system. Equations (3) and (4) express the low-pass filter and the proportional and derivative feedbacks with respect to *v*, respectively, where α, β, and $f_{f}$ are the proportional gain, derivative gain, and cut-off frequency of the filter, respectively. By suitably setting the control parameters, α, β, and $f_{f}$, we can realize sensorless self-excited oscillation for a highly sensitive viscometer. The system of Equation (3) plays a role not only as the filter for noise reduction, but also as the phase-shift controller proposed in [24,25]. This filter system is essential to the proposed viscometer because a change in the cut-off frequency $f_{f}$ qualitatively changes the dynamics of the whole fourth-order coupling system of Equations (1)–(4).

We introduce the representative time as $T_{r}=1/\omega_{0}=\sqrt{\frac{m}{k_{0}+k_{f}}}$, where $\omega_{0}$ is the natural frequency of the first mode of the cantilever. Using the dimensionless independent variable $t^{*}=t/T_{r}$, we obtain the dimensionless governing equations of the entire sensorless viscometer system as

(5) $$\begin{array}{c} \ddot{a}+2\gamma \dot{a}+a=-\frac{\tilde{\psi_{1}}}{\delta f_{f}}{\dot{v}}_{\mathrm{LPF}}-{\tilde{\psi }}_{1}v_{\mathrm{LPF}}, \end{array}$$

(6) $$\begin{array}{c} {\ddot{v}}_{\mathrm{LPF}}+\tilde{\beta }\delta f_{f}{\dot{v}}_{\mathrm{LPF}}-\tilde{\alpha }\delta f_{f}v_{\mathrm{LPF}}={\tilde{\psi }}_{2}\delta f_{f}\dot{a}, \end{array}$$

where [ $\dot{\;}$ ] denotes the derivative with respect to the dimensionless time $t^{*}$. The other dimensionless parameters are expressed as

(7) $$\begin{array}{c} \gamma =\frac{c_{0}+c_{f}}{2\sqrt{m\left(k_{0}+k_{f}\right)}},\;{\tilde{\psi }}_{1}=\frac{\psi }{k_{0}+k_{f}},\;{\tilde{\psi }}_{2}=\frac{\psi }{C_{p}},\;\tilde{\alpha }=\frac{\alpha }{C_{p}}\frac{m}{k_{0}+k_{f}},\;\tilde{\beta }=1-\frac{\beta }{C_{p}},\;\delta f_{f}=\sqrt{\frac{m}{k_{0}+k_{f}}}f_{f}, \end{array}$$

where γ is the damping ratio (since this damping ratio corresponds to the measured viscosity as described in Section 3.2, we sometimes call γ viscosity in the theoretical analysis); $\tilde{\psi_{1}}$ and $\tilde{\psi_{2}}$ are constant; and $\tilde{\alpha }$, $\tilde{\beta }$, and $\delta f_{f}$ are dimensionless control parameters, which are suitably set. The matrix forms of Equations (5) and (6) are

(8) $$\frac{d}{dt^{*}}\left[\begin{array}{c} a\\ \dot{a}\\ v_{\mathrm{LPF}}\\ {\dot{v}}_{\mathrm{LPF}} \end{array}\right]=A\left[\begin{array}{c} a\\ \dot{a}\\ v_{\mathrm{LPF}}\\ {\dot{v}}_{\mathrm{LPF}} \end{array}\right],$$

where

(9) $$A=\left[\begin{array}{cccc} 0 & 1 & 0 & 0\\ -1 & -\gamma & -{\tilde{\psi }}_{1} & -{\tilde{\psi }}_{1}/\delta f_{f}\\ 0 & 0 & 0 & 1\\ 0 & {\tilde{\psi }}_{2}\delta f_{f} & \tilde{\alpha }\delta f_{f} & -\tilde{\beta }\delta f_{f} \end{array}\right].$$

### 2.2. Dynamics of the Viscometer System

**Figure 3:**
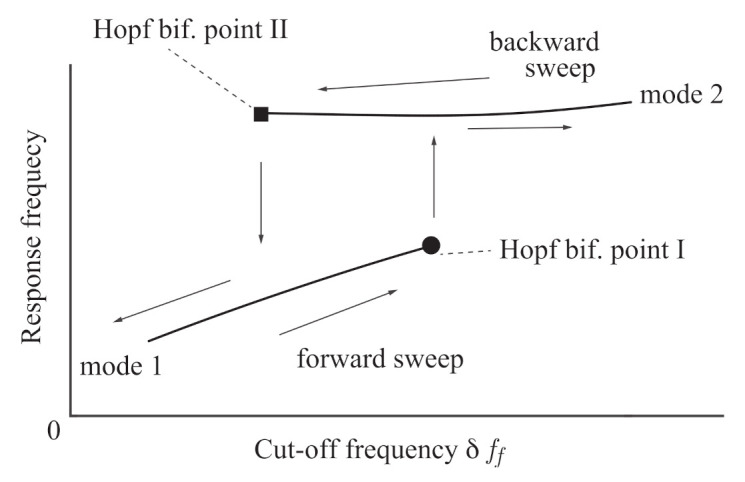
Schematic of the proposed method.

When the negative real part varies to positive, the self-excited oscillation is produced through Hopf bifurcation. The eigenvalues at the Hopf bifurcation point include a pair of conjugate pure imaginary values. Because Equations (8) and (9) are a fourth-order system, two different Hopf bifurcation points can exist at different values of the feedback control parameters, $\tilde{\alpha }$, $\tilde{\beta }$, and $\delta f_{f}$. In other words, two kinds of self-excited oscillations with different modes corresponding to lower and higher response frequencies can be produced in a parameter range. Figure 3 shows a schematic of the proposed method. We increase the control parameter $\delta f_{f}$ (forward sweep) from the state where a self-excited oscillation occurs with mode 1, which is related to the lower response frequency. The control parameter $\delta f_{f}$ reaches its value at Hopf bifurcation II. The other self-excited oscillation with mode 2, which is related to the higher response frequency, is not produced, but the original self-excited oscillation with mode 1 continues. We continue increasing $\delta f_{f}$ until it reaches Hopf bifurcation I, where the original self-excited oscillation with mode 1 stops and the self-excited oscillation with mode 2 is suddenly produced. Then, the response frequency jumps up to the higher response frequency related to mode 2. This self-excited oscillation is maintained by increasing $\delta f_{f}$ more.

From this state, we decrease $\delta f_{f}$ (backward sweep). Different from the forward sweep, the parameter value reaches first the value at the Hopf bifurcation I. The self-excited oscillation with mode 1 is not produced, but the self-excited oscillation with mode 2 continues. The control parameter $\delta f_{f}$ is increased further, until it reaches Hopf bifurcation II, where the self-excited oscillation with mode 2 stops and the self-excited oscillation with mode 1 is suddenly produced. Here, the response jumps down to its lower frequency.

The distance between the two Hopf bifurcation points, I and II, is the width of the hysteresis. As theoretically shown below, this distance depends on the viscosity. The values of $\delta f_{f}$ at the two bifurcation points are obtained by detecting the jumps in the response frequency. Thus, the hysteresis between Hopf bifurcation points I and II is used in the proposed sensorless viscometer. Because this behavior is similar to the bi-stable range and jump phenomenon in nonlinear dynamics, we call this hysteresis “bi-unstable.” Using the bi-unstable range makes measurement highly sensitive and easier because the jump in the response frequency can be easily detected.

We next find the conditions for the existence of this bi-unstable range.

The characteristic equation of *A* can be expressed as

(10) $$\begin{array}{c} \lambda^{4}+\left(2\gamma +\tilde{\beta }\delta f_{f}\right)\lambda^{3}+\left(-\tilde{\alpha }\delta f_{f}+2\gamma \tilde{\beta }\delta f_{f}+{\tilde{\psi }}_{1}{\tilde{\psi }}_{2}+1\right)\lambda^{2}+\delta f_{f}\left(-2\gamma \tilde{\alpha }+\tilde{\beta }+{\tilde{\psi }}_{1}{\tilde{\psi }}_{2}\right)\lambda -\tilde{\alpha }\delta f_{f}=0, \end{array}$$

where λ is an eigenvalue. Before analyzing the eigenvalue, we determine the condition for producing the Hopf bifurcation points to clarify how the measured viscosity γ affects these points. The condition for the control parameters at the Hopf bifurcation point can be derived by substituting λ=jω, where $j=\sqrt{-1}$, into Equation (10) to obtain

(11) $$\begin{array}{cc} \; & \omega^{4}-\left(-{\tilde{\alpha }}_{\mathrm{cr}}\delta f_{f-\mathrm{cr}}+2\gamma {\tilde{\beta }}_{\mathrm{cr}}\delta f_{f-\mathrm{cr}}+{\tilde{\psi }}_{1}{\tilde{\psi }}_{2}+1\right)\omega^{2}-{\tilde{\alpha }}_{\mathrm{cr}}\delta f_{f-\mathrm{cr}}=0, \end{array}$$

(12) $$\begin{array}{cc} \; & -\left(2\gamma +{\tilde{\beta }}_{\mathrm{cr}}\delta f_{f-\mathrm{cr}}\right)\omega^{2}+\delta f_{f-\mathrm{cr}}\left(-2\gamma {\tilde{\alpha }}_{\mathrm{cr}}+{\tilde{\beta }}_{\mathrm{cr}}+{\tilde{\psi }}_{1}{\tilde{\psi }}_{2}\right)=0, \end{array}$$

where ω is the dimensionless self-excited frequency. Equations (11) and (12) can be rewritten in the linear form

(13) $$\left[\begin{array}{ccc} \omega^{2}-1 & -2\gamma \omega^{2} & \omega^{2}\left(\omega^{2}-1\right)-{\tilde{\psi }}_{1}{\tilde{\psi }}_{2}\omega^{2}\\ 2\gamma & 1-\omega^{2} & 2\gamma \omega^{2} \end{array}\right]\left[\begin{array}{c} {\tilde{\alpha }}_{\mathrm{cr}}\\ {\tilde{\beta }}_{\mathrm{cr}}\\ 1/\delta f_{f-\mathrm{cr}} \end{array}\right]=\left[\begin{array}{c} 0\\ {\tilde{\psi }}_{1}{\tilde{\psi }}_{2} \end{array}\right].$$

This linear form shows that the Hopf bifurcation point and its corresponding frequency ω are not unique. This indicates that it is possible to realize bi-unstable states.

### 2.3. Proposed Highly Sensitive Viscosity Measurement Using the Bi-Unstable Range

#### 2.3.1. Analysis of the Root Locus

**Figure 4:**
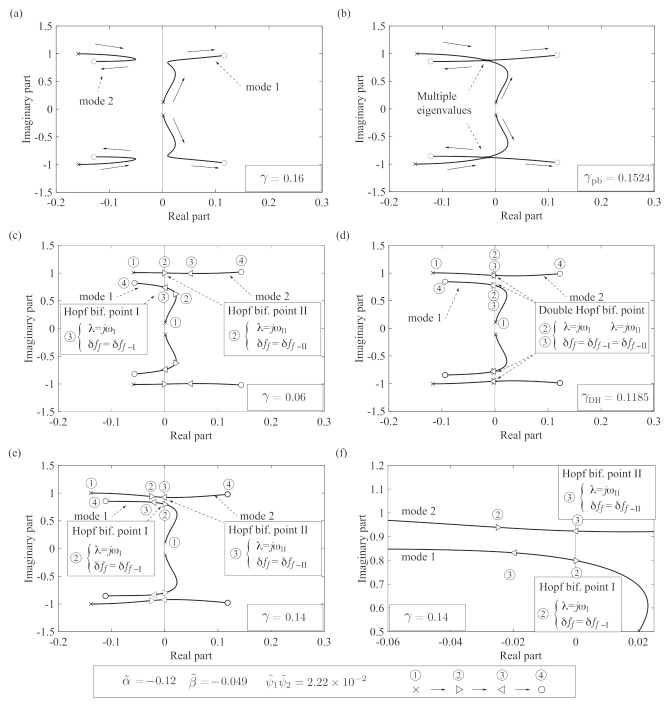
Numerical examples of the root locus when $\delta f_{f}$ varies from 0.1 to 6: (**a**) modes 1 and 2 are always unstable and stable, respectively; (**b**) multiple complex conjugate eigenvalues exist; (**c**) the bi-unstable range exists; (**d**) the bi-unstable range exists and includes a Double Hopf bifurcation point; (**e**) $\delta f_{f}$ has passed the Double Hopf bifurcation; (**f**) magnified view of (**e**). The root locus of (**c**), which is a possible case for viscosity measurement, transforms to that in (**a**) via the existence of multiple eigenvalues in (**b**), and to (**e**,**f**) via the Double Hopf bifurcation in (**d**).

Figure 4 shows the typical root loci obtained from Equation (10), where the filter parameter $\delta f_{f}$ is increased from 0.1 to 6 and the other control parameters are shown in the figure legend. The self-excited oscillation occurs when the eigenvalues are in the right half-plane.

First, we analyze the change from Figure 4a–c. Figure 4a shows a case in which the bi-unstable range does not exist because the eigenvalues corresponding to modes 1 and 2 are always in the unstable and stable plane, respectively. In this case, the self-excited oscillation of mode 1 is always produced regardless of the variation in the control parameter $\delta f_{f}$. Hence, the condition of Figure 4a is not suitable for the measurement. For the relatively low viscosity, the condition qualitatively changes as follows. When the measured viscosity γ decreases, the root locus in Figure 4a transforms to that in (c) via that in (b). At $\gamma =\gamma_{\mathrm{pb}}$, the root locus is expressed as in Figure 4b, and multiple complex conjugate eigenvalues are produced. When the measured viscosity γ decreases further, the root locus changes to that in Figure 4c. The eigenvalues move in ascending order of label number: ➀×→➁▹→➂◃→➃∘. As the control parameter $\delta f_{f}$ is swept forward in the cases of (b) and (c), the eigenvalues of mode 1 enter the stable plane when $\delta f_{f}$ reaches the value corresponding to Hopf bifurcation I, which is related to the lower response frequency. Before the eigenvalues of mode 1 enter the stable plane, the eigenvalues of mode 2 enter the unstable plane when the control parameter reaches Hopf bifurcation point II, which is related to the higher response frequency. The bi-unstable range is realized in this state. Hence, the condition of Figure 4c is suitable for the measurement. The above investigation shows that the viscosity $\gamma =\gamma_{\mathrm{pb}}$ in (b) is the boundary value of γ that realizes the bi-unstable range of (c), meaning it produces multiple complex conjugate eigenvalues.

Second, we investigate the state of Figure 4c in more detail. Under the forward sweep of the control parameter $\delta f_{f}$, the eigenvalues of mode 2 first enter the unstable plane (➁ ▷) at Hopf bifurcation point II ($\lambda =\pm j\omega_{\mathrm{II}}$) when $\delta f_{f}=\delta f_{f-\mathrm{II}}$. Second, as $\delta f_{f}$ increases further, the eigenvalues of mode 1 enter the stable plane (➂◁) at Hopf bifurcation point I ($\lambda =\pm j\omega_{I}$) when $\delta f_{f}=\delta f_{f-I}$. This indicates that the bi-unstable range exists when $\delta f_{f-I}>\delta f_{f-\mathrm{II}}$. These values of $\delta f_{f-I}$ and $\delta f_{f-\mathrm{II}}$ are endpoints of the bi-unstable range, representing the width of the hysteresis. The proposed sensorless viscometer is based on measuring the difference between these endpoints depending on the viscosity. Here, we consider the change of the root locus for much lower γ referring to the change from (c) to (e). This bi-unstable range monotonically shrinks as the viscosity γ increases, and becomes zero at $\gamma =\gamma_{\mathrm{DH}}$, where the root locus is expressed as in (d). In this situation, the eigenvalues of both modes become a pair of pure imaginary eigenvalues at the same parameter value. This situation is called a Double Hopf bifurcation point [32,33].

Figure 4e shows the case when the viscosity γ is higher than $\gamma_{\mathrm{DH}}$ at the Double Hopf bifurcation point, and (f) is its magnified view. Unlike the case of (c), the eigenvalues of mode 2 enter the unstable plane (➂ ▷) after the eigenvalues of mode 1 enter the stable plane (➁◁). This indicates that both modes are stable between ➁ and ➂, and the bi-unstable range does not exist, meaning $\delta f_{f-I}<\delta f_{f-\mathrm{II}}$. Hence, the conditions of Figure 4e,f are not suitable for the measurement.

As the viscosity γ increases, the bi-unstable range (the difference $\delta f_{f-I}-\delta f_{f-\mathrm{II}}$) monotonically shrinks and disappears at the Double Hopf bifurcation point ($\gamma =\gamma_{\mathrm{DH}}$) as shown in Figure 4c–f. From the next section, we analyze the relationship between $\gamma_{\mathrm{pb}}$ and the control parameter $\delta f_{f}$ and examine the endpoints $\delta f_{f-I}$ and $\delta f_{f-\mathrm{II}}$.

#### 2.3.2. Condition for the Multiple Eigenvalues

First, we find the condition for the matrix *A* of Equation (8) to have two multiple complex conjugate eigenvalues as shown in Figure 4b. The characteristic equation can be written as

(14) $$\begin{array}{c} {\left(\lambda -\lambda_{m}\right)}^{2}{\left(\lambda -{\bar{\lambda }}_{m}\right)}^{2}=0, \end{array}$$

where $\lambda_{m}$ is a multiple complex eigenvalue. Comparing the coefficients of Equation (14) with those of Equation (10) leads to the following equations:

(15) λ3:Re[λm]=2γpb+β˜δff−pb,

(16) λ2:2λm2+4Re[λm]2=−α˜δff−pb+2γpbδff−pb+ψ˜1ψ˜2+1,

(17) λ2:−4Re[λm]λm2=δff−pb−2γpbα˜+β˜+ψ˜1ψ˜2,

(18) $$\begin{array}{c} \lambda^{0}:\;{\left|\lambda_{m}\right|}^{4}=-\tilde{\alpha }\delta f_{f-\mathrm{pb}}, \end{array}$$

where Re[$\lambda_{m}$] denotes the real part of $\lambda_{m}$. To determine the parameters $\delta f_{f}$ and γ that produce the multiple complex conjugate eigenvalues, we eliminate $\lambda_{m}$ to obtain

(19) $$\begin{array}{c} \left(\frac{1}{2}\tilde{\beta }+\gamma_{\mathrm{pb}}\right)\left(\frac{{\tilde{\beta }}^{2}\delta f_{f-\mathrm{pb}}}{4}+\gamma_{\mathrm{pb}}^{2}+\tilde{\alpha }\delta f_{f-\mathrm{pb}}+\tilde{\beta }\delta f_{f-\mathrm{pb}}\gamma_{\mathrm{pb}}-{\tilde{\psi }}_{1}{\tilde{\psi }}_{2}-1\right)\\ \;=\delta f_{f-\mathrm{pb}}\left(2\gamma_{\mathrm{pb}}\tilde{\alpha }-\tilde{\beta }-{\tilde{\psi }}_{1}{\tilde{\psi }}_{2}\right), \end{array}$$

(20) $$\begin{array}{cc} \; & 2\sqrt{-\tilde{\alpha }\delta f_{f-\mathrm{pb}}}+\frac{{\tilde{\beta }}^{2}\delta f_{f-\mathrm{pb}}}{4}+\gamma_{\mathrm{pb}}-\tilde{\beta }\delta f_{f-\mathrm{pb}}\gamma_{\mathrm{pb}}+\tilde{\alpha }\delta f_{f-\mathrm{pb}}-{\tilde{\psi }}_{1}{\tilde{\psi }}_{2}-1=0, \end{array}$$

where subscript ‘${}_{\mathrm{pb}}$’ denotes the parameter values that produce the multiple complex conjugate eigenvalues. When the parameters $\delta f_{f}$ and γ satisfy Equations (19) and (20), the multiple complex conjugate eigenvalues appear as shown in Figure 4b. If the viscosity γ is greater than the boundary value $\gamma_{\mathrm{pb}}$, the eigenvalues of one mode become always unstable; the root locus in this situation is shown in Figure 4a.

#### 2.3.3. Derivation of the Endpoints of the Bi-Unstable Range

To derive the values of $\delta f_{f-I}$ and $\delta f_{f-\mathrm{II}}$ at the endpoints in Figure 4c–f, we analyze Equations (11)–(13). Solving Equation (11) for $\omega^{2}$ yields two self-excited oscillation frequencies at the Hopf bifurcation points:

(21) $$\begin{array}{cc} \omega_{I}^{2} & =\frac{r_{2I}-\sqrt{r_{2I}^{2}-4r_{0I}}}{2}≃\frac{r_{0I}}{r_{2I}},\\ \; & \mathrm{where}\;r_{2I}=-{\tilde{\alpha }}_{\mathrm{cr}}\delta f_{f-I}+2\gamma {\tilde{\beta }}_{\mathrm{cr}}\delta f_{f-I}+{\tilde{\psi }}_{1}{\tilde{\psi }}_{2}+1,\;r_{0I}=-{\tilde{\alpha }}_{\mathrm{cr}}\delta f_{f-I}, \end{array}$$

(22) $$\begin{array}{cc} \omega_{\mathrm{II}}^{2} & =\frac{r_{2\mathrm{II}}+\sqrt{r_{2\mathrm{II}}^{2}-4r_{0\mathrm{II}}}}{2}≃r_{2\mathrm{II}},\\ \; & \mathrm{where}\;r_{2\mathrm{II}}=-{\tilde{\alpha }}_{\mathrm{cr}}\delta f_{f-\mathrm{II}}+2\gamma {\tilde{\beta }}_{\mathrm{cr}}\delta f_{f-\mathrm{II}}+{\tilde{\psi }}_{1}{\tilde{\psi }}_{2}+1,\;r_{0\mathrm{II}}=-{\tilde{\alpha }}_{\mathrm{cr}}\delta f_{f-\mathrm{II}}. \end{array}$$

The conditions $\frac{4r_{0I}}{r_{2I}^{2}}<<1$ and $\frac{4r_{0\mathrm{II}}}{r_{2\mathrm{II}}^{2}}<<1$ are assumed, and $\omega_{I}$ and $\omega_{\mathrm{II}}$ are the response frequencies at Hopf bifurcation points I and II, respectively, as shown in Figure 4c–e. Substituting Equations (21) and (22) into Equation (12) and solving for $\delta f_{f-I}$ and $\delta f_{f-\mathrm{II}}$ yields

(23) $$\begin{array}{c} \delta f_{f-I}=\frac{-2{\tilde{\psi }}_{1}{\tilde{\psi }}_{2}{\tilde{\alpha }}_{\mathrm{cr}}\gamma +\left({\tilde{\beta }}_{\mathrm{cr}}+{\tilde{\psi }}_{1}{\tilde{\psi }}_{2}\right)\left(1+{\tilde{\psi }}_{1}{\tilde{\psi }}_{2}\right)}{4{\tilde{\alpha }}_{\mathrm{cr}}{\tilde{\beta }}_{\mathrm{cr}}\gamma^{2}-2\left({\tilde{\beta }}_{\mathrm{cr}}^{2}+{\tilde{\psi }}_{1}{\tilde{\psi }}_{2}{\tilde{\beta }}_{\mathrm{cr}}+{\tilde{\alpha }}_{\mathrm{cr}}^{2}\right)\gamma +{\tilde{\psi }}_{1}{\tilde{\psi }}_{2}{\tilde{\alpha }}_{\mathrm{cr}}}, \end{array}$$

(24) $$\begin{array}{c} \delta f_{f-\mathrm{II}}=\frac{4{\tilde{\beta }}_{\mathrm{cr}}\gamma^{2}-{\tilde{\psi }}_{1}{\tilde{\psi }}_{2}\left(1-{\tilde{\beta }}_{\mathrm{cr}}\right)+\sqrt{D}}{-2{\tilde{\beta }}_{\mathrm{cr}}\left(2{\tilde{\beta }}_{\mathrm{cr}}\gamma -{\tilde{\alpha }}_{\mathrm{cr}}\right)}, \end{array}$$

where $D={\left[4{\tilde{\beta }}_{\mathrm{cr}}\gamma^{2}-{\tilde{\psi }}_{1}{\tilde{\psi }}_{2}\left(1-{\tilde{\beta }}_{\mathrm{cr}}\right)\right]}^{2}-8{\tilde{\beta }}_{\mathrm{cr}}\left(2{\tilde{\beta }}_{\mathrm{cr}}\gamma -{\tilde{\alpha }}_{\mathrm{cr}}\right)\left(1+{\tilde{\psi }}_{1}{\tilde{\psi }}_{2}\right)\gamma$. Equations (23) and (24) respectively denote the endpoint values of the control parameter, $\delta f_{f-I}$ and $\delta f_{f-\mathrm{II}}$, that produce the Hopf bifurcations $\lambda =\pm j\omega_{I}$ and $\lambda \;\pm j\omega_{\mathrm{II}}$ in Figure 4c–f.

### 2.4. Summary of the Proposed Method

**Figure 5:**
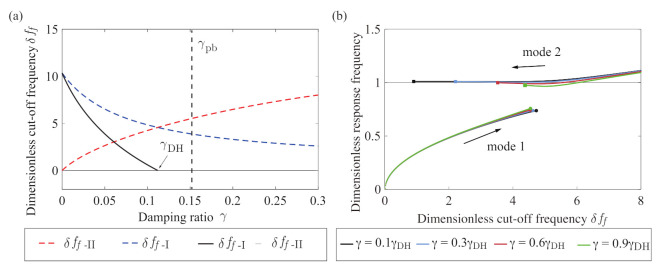
Numerical example of the relationship between the viscosity γ and the bi-unstable range. (**a**) Change in the bi-unstable range obtained from Equations (23) and (24). The black dashed line denotes the boundary value that produces the multiple eigenvalues in Figure 4b obtained by solving Equations (19) and (20). The blue and red dashed lines show the control parameters, Equations (23) and (24), respectively, and the black line shows their difference. $\gamma_{\mathrm{pb}}$ and $\gamma_{\mathrm{DH}}$ correspond to the root loci of Figure 4b,d, respectively. (**b**) Response frequency of the sensorless self-excited oscillation. The black, light blue, red, and light green lines show the expected dimensionless response frequency ω when $\gamma =0.1\gamma_{\mathrm{DH}}$, $\gamma =0.3\gamma_{\mathrm{DH}}$, $\gamma =0.6\gamma_{\mathrm{DH}}$, and $\gamma =0.9\gamma_{\mathrm{DH}}$, respectively, where $\gamma_{\mathrm{DH}}$ is the same as in (**a**). The circle and square markers show the Hopf bifurcation points for modes 1 and 2. The difference in $\delta f_{f}$ of the square and circle markers for each γ is the hysteresis width. The hysteresis depends on viscosity γ.

In this section, we summarize the proposed method by showing a numerical example of the endpoints. Figure 5a shows a numerical example of a change in the bi-unstable range depending on the viscosity γ obtained from Equations (23) and (24). This describes the changes in the endpoints of the bi-unstable range in the root loci of Figure 4c–e. Furthermore, $\gamma =\gamma_{\mathrm{pb}}$, which is the limit condition in Figure 4b, is obtained from Equations (19) and (20) and is shown with the black dashed line. The parameter values are the same as those of Figure 4. Because $\gamma_{\mathrm{pb}}>\gamma_{\mathrm{DH}}$ is satisfied in Figure 5, the bi-unstable range disappears at the Double Hopf bifurcation point $\gamma =\gamma_{\mathrm{DH}}$. If $\gamma_{\mathrm{pb}}<\gamma_{\mathrm{DH}}$, the bi-unstable range is $0<\gamma <\gamma_{\mathrm{pb}}$.

The values of $\delta f_{f-I}$ and $\delta f_{f-\mathrm{II}}$ decrease and increase monotonically with increasing viscosity γ, respectively. The rates of change in $\delta f_{f-\mathrm{II}}$ and $\delta f_{f-I}$ with respect to γ are smaller than that of the difference $\delta f_{f-I}-\delta f_{f-\mathrm{II}}$. Because these rates are related to the sensitivity, using two Hopf bifurcations provides higher sensitivity. This changing rate of the difference in endpoints, $\delta f_{f-I}-\delta f_{f-\mathrm{II}}$, with increasing viscosity γ can be changed by setting $\tilde{\alpha }$ and $\tilde{\beta }$. The above discussion gives the method to appropriately set the parameters of the measurement system depending on the measured viscosity range.

In practical measurements, only the response frequency corresponding to the imaginary part of the eigenvalue can be measured. Figure 5b shows the change in the dimensionless response frequency depending on the control parameter $\delta f_{f}$. The figure shows the results for four different viscosities: $\gamma =0.1\gamma_{\mathrm{DH}}$, $\gamma =0.3\gamma_{\mathrm{DH}}$, $\gamma =0.6\gamma_{\mathrm{DH}}$, and $\gamma =0.9\gamma_{\mathrm{DH}}$, where $\gamma_{\mathrm{DH}}$ denotes γ at the Double Hopf bifurcation point. The bi-unstable range appears between the square (□) and circle (∘) markers and decreases with increasing γ. When the control parameter $\delta f_{f}$ is set at a point □ or ∘, the jump occurs depending on the sweep direction. By measuring the jump occurring at the markers □ and ∘, we experimentally detect the endpoints of the hysteresis (the hysteresis width) depending on viscosity.

## 3. Experiment

We verified the efficiency of the method constructed above by experimentally measuring viscosity using the proposed viscometer based on sensorless self-excited oscillation.

### 3.1. Experimental Setup and Basic Properties

**Figure 6:**
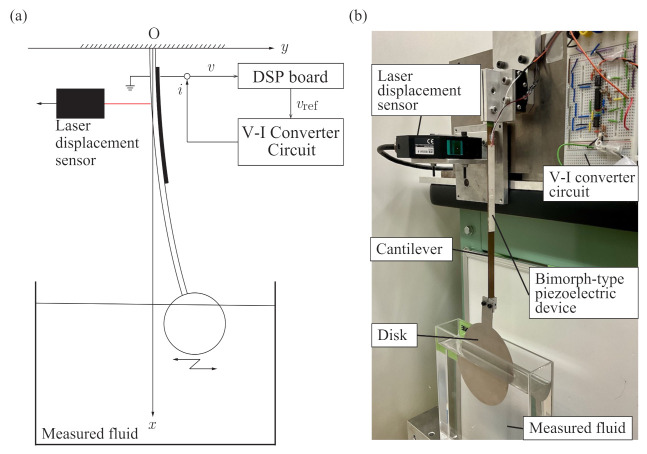
Experimental setup. (**a**) Signal flow for producing the self-excited oscillation. (**b**) Photograph of the sensorless viscometer system.

Figure 6a,b show the signal flow for the measurement system and a photograph of the experimental equipment, respectively. A bimorphic piezoelectric device (100 × 5 × 0.5 mm, Fuji Ceramics Corp. (Fujinomiya, Japan), c91) was attached to a phosphor bronze macro-cantilever (207 × 5 × 0.7 mm). A thin rigid disk (diameter: 100 mm; thickness: 1.0 mm) was attached at the free end of the cantilever to measure the viscosity of sample fluids. The voltage *v* between the terminals of the piezoelectric device was measured and analyzed using a fast Fourier transform (FFT). The control input *i* to produce self-excited oscillation was calculated according to Equations (3) and (4) on a digital signal processing (DSP) board (DS1104, dSPACE GmbH, Paderborn, Germany) and applied through a V-I converter circuit. The response displacement was measured using a laser displacement sensor (LK-G35A, Keyence Corp., Osaka, Japan) to preliminary collect the fundamental dynamics of the cantilever with the piezoelectric device, but was not used for feedback control. We used three sample fluids: water and two different standard hydrocarbon liquids (JS5 and JS2.5, Nippon Grease Corp., Yokohama, Japan). The accuracies of the properties of JS5 and JS2.5 comply with Japanese Industrial Standard (JIS) Z 8809 (https://www.jisc.go.jp/app/jis/general/GnrJISSearch.html (accessed on 5 February 2021)). Their nominal viscosities are shown in Table 1. The other parameters are shown in Table 2. We further assumed that the natural frequency shift due to the fluid could be neglected because the bending stiffness of the cantilever was sufficiently higher than that added by the measured fluid.

**Figure 7:**
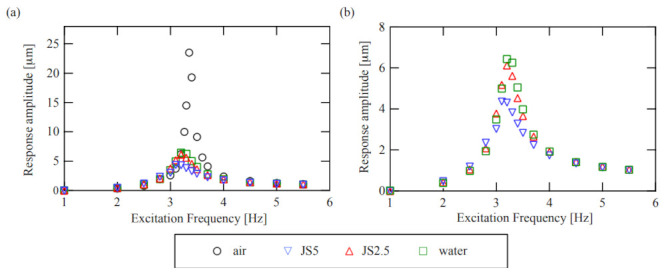
Frequency response curves under external harmonic excitation measured with the laser displacement sensor: (**a**) overall view; (**b**) magnified view. The black markers show the characteristic of the resonator itself. The green, red, and blue markers show the characteristics when the thin disk is immersed in the water and sample fluids with viscosities of 1.94 mPa s and 4.06 mPa s, respectively.

First, in Figure 7a,b, we compare with a conventional method by showing the frequency response under the external harmonic excitation, which was measured with the displacement sensor for each condition. The black circles show the frequency response curve of the resonator itself, i.e., the cantilever with the piezoelectric device, which is labeled “air.” The green, red, and blue markers show the frequency response curves for water, JS2.5, and JS5, respectively. While the Q-value of the resonator itself is much greater than those of the three samples, the Q-values of the three samples do not seem different from each other. The viscosities of these three sample fluids are almost the same and cannot be determined from the frequency response curves under external excitation in the conventional method.

### 3.2. Viscosity Measurement via the Proposed Method

The experimental procedure consisted of three steps. The first step was producing the sensorless self-excited oscillation. This step was setting the feedback gains so that the self-excited oscillation occurred with a fixed cut-off frequency $f_{f}$. The second step was varying $f_{f}$ and seeking two Hopf bifurcation points. By detecting the response frequency while varying $f_{f}$, the Hopf bifurcation points can be easily found from the jumps up and down in the response frequency if the bi-unstable range exists. The third step was calculating the magnitude of the bi-unstable range, which is the difference in values of $f_{f}$ for the two Hopf bifurcation points (the hysteresis width). We carried out these measurements using two fixedPD (proportional-derivative) feedback controllers (Case A and Case B), whose parameters are shown in Table 3.

**Figure 8:**
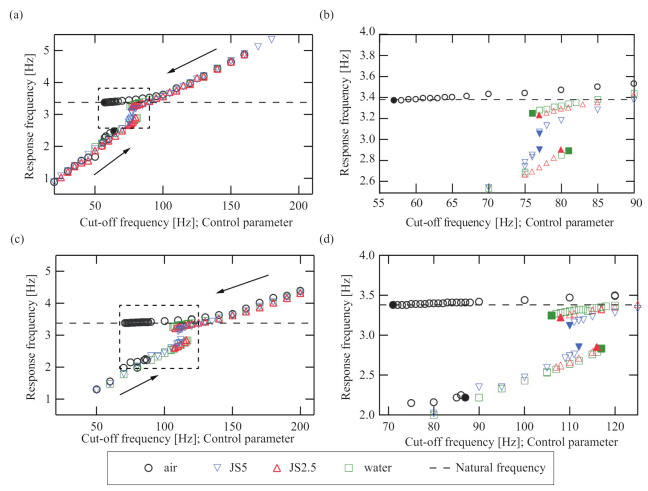
Response frequency under the sweep of the control parameter $\delta f_{f}$. (**a**–**d**) show the responses for Case A and Case B, respectively. (**b**,**d**) are the magnified views of (**a**,**c**), respectively. The black, blue, red, and green markers show the response frequency when the disk is not immersed in fluid, is immersed in JS5, JS2.5, and water, respectively. For all conditions, the response frequencies are divided into upper and lower branches. The response frequencies on the lower branch increase continuously with increasing cut-off frequency $f_{f}$ and jump up to the upper branch at each Hopf bifurcation point depending on the viscosity, which is denoted by colored makers. Those on the upper branch decrease continuously with decreasing cut-off frequency $f_{f}$ and jump down to the lower branch at the other Hopf bifurcation point depending on the viscosity of the sample fluid, which is denoted by colored markers.

Figure 8 shows the experimental plot of the response frequency with varying cut-off frequency $f_{f}$ corresponding to Figure 5b. Figure 8a,c show the response frequencies for cases A and B, respectively, and (b) and (d) show the magnified views of the dashed squares in (a) and (c), respectively. The black, blue, red, and green markers show the responses of the thin rigid disk when it is immersed in air, JS5, JS2.5, and water, respectively, and the colored markers denote the jump points at the Hopf bifurcations. The lower and upper branches in both results of Figure 8 (cases A and B) are experimentally detected from the cut-off frequency $f_{f}$. As for the theoretical response in Figure 5b, the bi-unstable ranges exist in the region between the colored markers. The theoretical result shows that both modes can be destabilized simultaneously, but the experimental results do not show such a destabilization. It is ensured in the experiments that one of the self-excited oscillations even in the bi-unstable range is excited and hysteresis is produced. The theoretical prediction that the two modes are not simultaneously destabilized requires nonlinear stability analysis that is left for future work. In both cases A and B, the magnitude of the bi-unstable range (the hysteresis width) monotonically shrinks with increasing viscosity of the immersed fluid. This characteristic is shown in Figure 5a.

**Figure 9:**
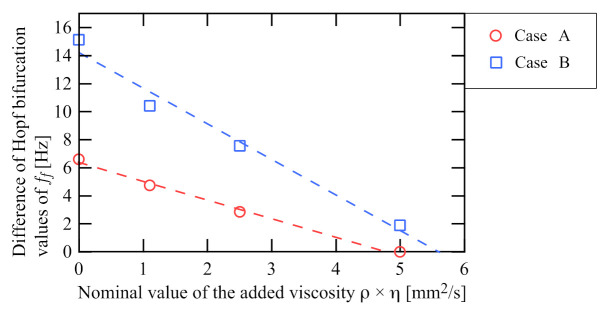
Relationship between the width of the bi-unstable range and added kinematic viscosity ρ×η. The red and blue markers denote the magnitude of the bi-unstable range obtained from Figure 8. The dashed lines are their linear fits. Both monotonically decrease with increasing measured kinematic viscosity. The gradient of Case B is greater than that of Case A.

Next, Figure 9 shows the experimentally obtained bi-unstable ranges depending on the kinematic viscosity ρ×η, where the red and blue markers are the results for cases A and B, respectively, and the dashed lines denote their linear fits. This bi-unstable range directly depends on the damping ratio γ, not viscosity η. However, the measurement of the damping ratio γ corresponds to that of the kinematic viscosity ρ×η because the damping ratio γ is a function of the kinematic viscosity ρ×η under the condition that the response frequency of the vibrational viscometer is approximately constant as in the present experiments: Figure 8 (for detail, see Appendix A). Accurate estimation of density ρ is much easier because static measurement methods can be used as a pycnometer based on Archimedes’ principle [34]. For these reasons, the proposed measurement method based on the bi-instability can be regarded as a measurement for viscosity. Because this plot also agrees well with the theoretical result in Figure 5a, it is fair to say that the proposed sensorless viscometer measures the viscosity.

### 3.3. Evaluation and Discussion

**Figure 10:**
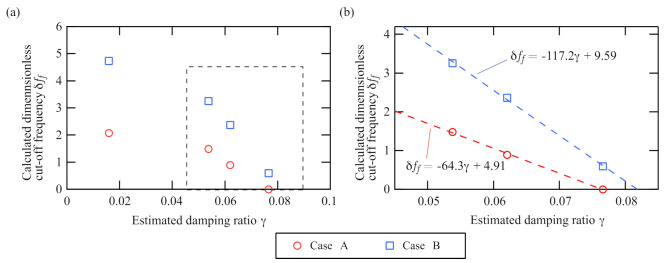
Nondimensionalized experimental results of Figure 9: (**a**) overall view; (**b**) magnified view. The horizontal and vertical axes show the damping ratio estimated from the preliminary experiments and the dimensionless cut-off frequency, respectively. The dimensionless cut-off frequency is the cut-off frequency divided by the natural frequency of the cantilever. The dashed lines in (**b**) are the linear fits of the markers.

Finally, we examine the sensitivity of the experimental results. The experimental results in Figure 9 show the characteristic of the bi-unstable range (the hysteresis width) depending on the viscosity of the fluid in which the disk at the tip of the cantilever is immersed. This is enough to confirm whether the proposed principle works as a sensor, but Figure 9 is not suitable for validation because the result is affected by the viscosity owing to the cantilever itself and the magnitude of the natural frequency. Because the proposed method is constructed using the dimensionless Equations (8) and (9), the experimental results need to be nondimensionalized to verify their sensitivity fairly. To derive the dimensionless results, we estimate the damping ratio from preliminary experiments that measured the free decay response of the cantilever using the laser displacement sensor. Figure 10 shows the nondimensionalized experimental results. The horizontal and vertical axes are the estimated damping ratio and the dimensionless cut-off frequency of the filter ($2\pi f_{f}/\omega_{0}$ in Figure 9), respectively.

First, we validate the sensitivity by comparing the experimental results of the proposed method with those of the previous method, which uses self-excited oscillation with a sensor [20,21]. The previous viscometer, based on feedback control with a displacement sensor, determines the viscosity from the critical feedback control parameter that exactly cancels the viscosity [20,21]. The measured feedback control parameter is proportional to the viscosity with a proportionality ratio of 1. In contrast, the dimensionless control parameter $\delta f_{f}$ in the proposed method is approximately proportional to the viscosity with a proportionality ratio greater than 1 as shown in Figure 10b. In particular, the ratio in the result for Case B, which is denoted by blue, is approximately 117 because its linear fit is $\delta f_{f}=-117.2\gamma +9.59$.

The proposed method needs only to detect the jump points, which are indicated in Figure 5b and Figure 8, unlike other methods that use frequency analysis or phase detection. This method makes measurement easier in two ways: no displacement sensors are used, and no complicated analysis such as phase detection or highly accurate FFT is needed. Although there is a limitation due to the condition for the bi-unstable range as stated in Section 2.3, the proposed viscometer based on sensorless self-excited oscillation provides highly sensitive and easy viscosity measurement.

## 4. Conclusions

In this study, we have realized a sensorless viscometer by producing self-excited oscillation based on the interaction between the mechanical dynamics of a cantilever and the electrical dynamics of a circuit including a piezoelectric device. To enhance the sensitivity and ease of viscosity measurement, we proposed measurement based on bi-instability and on the jump occurring at the endpoints while the cut-off frequency is varied. The efficiency of the proposed method was demonstrated via practical viscosity measurements using a macro-scale cantilever with a bimorphic piezoelectric device. We measured the viscosity according to the theoretically proposed method of detecting the filter gains occurring at jumps in the response frequency. The results show the sensitivity of the proposed method is higher than that of the previous feedback method with a displacement sensor. Although the proposed method is limited to the measurement in the bi-unstable region, the magnitude of the region can be changed by the setting of the feedback gains. The proposed method features its high-usability, i.e., the proposed method does not require any detection and complex analysis of the response displacement or velocity. 

## Figures and Tables

**Table 1:** Nominal properties of the sample fluids. The accuracies for JS2.5 and JS5 comply with Japanese Industrial Standard (JIS) Z 8809.

Sample Label	JS5	JS2.5	Water
density ρ [kg/m${}^{3}$]	8.130 $\times \;10^{2}$	7.728 $\times \;10^{2}$	1 $\times \;10^{3}$
viscosity η [mPa s]	4.067	1.936	1

**Table 2:** Parameter values of the viscometer system.

Symbol	Value	Unit
ψ	3.27 $\times \;10^{-3}$	N/V
*m*	4 $\times \;10^{-2}$	kg
$\omega_{0}$	10.5	rad/s
$C_{p}$	$1.86\times \;10^{-7}$	F

**Table 3:** Experimental conditions.

Condition Label	Case A	Case B
Proportional gain α [A/V]	−4.46 $\times \;10^{-8}$	−4.46 $\times \;10^{-8}$
Derivative gain β [A/Vs]	1.47 $\times \;10^{-7}$	1.25 $\times \;10^{-7}$

**Figure A1:**
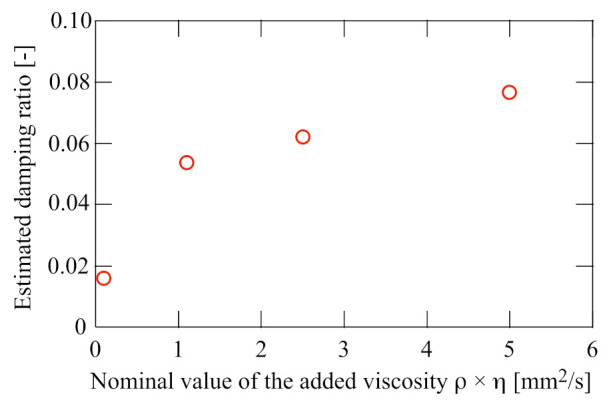
Experimentally derived characteristic between the damping ratio γ and the kinematic viscosity ρ×η.

## Data Availability

Not applicable.

## Appendix A

We show the characteristic between the damping ratio γ and measured viscosity η. The damping ratio γ is expressed as

(A1) $$\begin{array}{c} \gamma =c_{\gamma }\sqrt{\rho \;\eta }, \end{array}$$

where the coefficient $c_{\gamma }$ is derived from the first mode function of the cantilever $\Phi_{1}$ and the area of the oscillating disk immersed in the sample fluid [21,30]. On the other hand, an experimentally obtained relationship between the damping ratio γ and the kinematic viscosity ρ×η is shown in Figure A1. Figure A1 qualitatively agrees with Equation (A1): the reason why the intercept is not zero is that the damping of the cantilever itself is not considered in Equation (A1).
